# Association between Maternal and Child Dietary Diversity: An Analysis of the Ghana Demographic and Health Survey

**DOI:** 10.1371/journal.pone.0136748

**Published:** 2015-08-25

**Authors:** Dickson Abanimi Amugsi, Maurice B. Mittelmark, Abraham Oduro

**Affiliations:** 1 Navrongo Health Research Centre, Navrongo, Upper East Region, Ghana; 2 Department of Health Promotion and Development, Faculty of Psychology, University of Bergen, Bergen, Norway; University of Bath, UNITED KINGDOM

## Abstract

**Objective (s):**

This study examined the association between maternal and child dietary diversity in a population-based national sample in Ghana.

**Methods:**

The data for this analysis are from the 2008 Ghana Demographic and Health Survey. We used data obtained from 1187 dyads comprised of mothers’ ages 15–49 and their youngest child (ages 6–36 months). Maternal and child dietary diversity scores (DDS) were created based on the mother’s recall of her own and her child’s consumption of 15 food groups, during the 24 hours prior to the in-home survey. The same food groups were used to compose both maternal and child DDS. Linear regression was used to assess the relationship between the predicted outcome – child DDS -- and maternal DDS, taking into account child age and sex, maternal factors (age, education, occupation, literacy, empowerment, number of antenatal visits as an indicator of health care use), household Wealth Index, and urban/rural place of residence.

**Results:**

There was a statistically significant positive association between child and maternal DDS, after adjusting for all other variables. A difference of one food group in mother’s consumption was associated with a difference of 0.72 food groups in the child’s food consumption (95% CI: 0.63, 0.82). Also, statistically significant positive associations were observed such that higher child DDS was associated with older child age, and with greater women’s empowerment.

**Conclusions:**

The results show a significant positive association between child and maternal DD, after accounting for the influence of child, maternal and household level factors. Since the likely path of influence is that maternal DDS impacts child DDS, public health efforts to improve child health may be strengthened by promoting maternal DDS due to its potential for a widened effect on the entire family.

## Introduction

Nutritious foods and diverse diets in sufficient quality and quantity are essential for children to meet their nutrient needs and support growth. This is especially important during the first 1000 days of the child’s life, a critical window for the promotion of optimal child growth, health and development [1,2]. Dietary diversity (DD), defined as the sum of food groups consumed over a period of 24 hours, has been documented as a valid and reliable indicator of dietary adequacy of young children [3–8]. Therefore, DD is a reasonably easy-to-measure proxy variable for young children’s nutrient intake [9–14], and the World Health Organization (WHO) uses DD as one of the key indicators to assess child feeding practices [15]. In child health promotion, emphasizing DD helps focus families’ attention on what is in the family pot, rather than on the complex details of dietetics.

Thus, there is growing appreciation of the importance of DD for child health, and calls for research to illuminate the ways in which child DD can be promoted [4, 13, 16]. Since mothers usually play the most vital role in the healthcare of their children, research is needed to illuminate maternal factors that might promote child DD. One of the most proximal maternal factors may be maternal DD, following a logic that all family members eat from the same family pot. Yet not feeding small children from the family pot is common. Beliefs about which foods are appropriate for small children have long been observed to determine child diet as much as food availability and socioeconomic status [17]. It is fairly common that children and their mothers do not have the same meal composition [18]. Cultural determinants of child diet can exclude foods that are available in the home and that other family members eat. In previous research in rural Vietnam, many young children received little of the protein (rice paddy crab, fish and shrimp) that was otherwise ubiquitous in the family pot, because crustaceans were deemed by their caregivers to be inappropriate food for young children [19]. Quite aside from dietary concerns, Zeitlin [20] describes a time in Nigeria when parents used food restriction to develop children’s moral character. Oniang’o and Komokoti’s [21] description of food taboos in Eastern Kenya illustrates the multitude of factors that can underlie variation in the feeding of various family members:

“…children were not allowed to eat offal as it was believed that this would interfere with their growth. Pregnant women were not allowed to take bitter substances such as juices from medicinal trees. They too were not allowed to eat offal… Among the Embu, pregnant women did not eat food that included beans, as it was believed that this would cause constipation… Some ethnic groups in Western Kenya have traditionally prohibited pregnant women from eating eggs even where there is an abundant supply. The reason often given is that if women and children were allowed to eat eggs, there would be no chickens. In the same communities, chicken meat is a delicacy reserved for men and guests only. Among traditional Maasai, a pregnant woman is advised to avoid fatty foods, and instead drink cow’s blood, sour milk and lots of water, and vomit following a heavy meal; the intent here is to keep the baby small at birth and thus ensure safe delivery for both mother and baby.” [p. 94].

The studies just mentioned warn against assuming that all members of a group that is fed from the same kitchen share the same pot. Yet, a few studies, mostly from the USA, do indicate that what mothers eat is strongly associated with what their children eat. In the Midwestern USA, the diets of mothers and of their children were assessed at 6 months of age and again at 14 months; at both time points, the diet quality of the mother was highly concordant with the child’s diet [22]. In the rural north-central USA, maternal nutritional adequacy was observed to be poorer than the nutritional adequacy of their children, yet their dietary quality was correlated; mothers who did not eat at least one serving of fruits and vegetables had toddlers with nutrition inadequacy [23]. In a North-eastern urban area in the USA, mothers’ intake of fruits, vegetables and snack foods was significantly related to children’s food intake at ages 6 to 18 months [24]. In a nationally representative sample, the diets of female heads of households were strongly related to the diets of children in the household [25]. In the UK, studies of mother-child diet quality showed results similar to the USA [26]. Only two relevant non-Western publications were found in the literature. Children from Bangladesh, Vietnam and Ethiopia had levels of dietary diversity strongly related to maternal dietary diversity [27]. In an analysis of USAID’s Infant and Young Child Nutrition Project using the demographic and health survey (DHS) data from Ghana, Haiti and Cambodia, there were marked similarities between the ‘dietary fingerprints’ of mothers and their children [28].

In light of the mix of viewpoints and findings just reviewed, and with just one published analysis using Ghana data, no credible conclusion can yet be reached about the association of the Ghanaian child diet to family diet generally, and to maternal diet in particular. This is an issue of public health significance in Ghana. If the entire family eats from the same pot, health education and nutrition policy may prudently be formulated for entire groups sharing the same kitchen pot (nuclear or extended families or larger groups). However, if small children are fed tailored diets, special public health efforts may be needed to ensure that the quality of their feeding meets their essential growth needs. Infant and Young Child Nutrition Project investigators have summarised the state of knowledge succinctly: in lower income countries, the diets of women are often overlooked, and research is needed to document the extent to which mothers’ diets reflect the diets of the family members, particularly the children [28].

Besides the possibility of maternal diet, a child’s dietary diversity could be expected to vary according to the child’s age, the number of children in the household, the household’s socio-economic status, and/or maternal education level [27]. Other factors that are important determinants of child DD include maternal empowerment [29], maternal occupation, and maternal use of health care services [30].

Given the background just described, the objective of this study was to examine the association between child and maternal DD in Ghana, with adjustment for a range of other factors that might affect child diet.

## Materials and Methods

### Data sources and study sample

This study used data from the 2008 Ghana Demographic and Health Survey (DHS). The DHS project obtained ethical clearance from the Ghana Health Service ethical review committee before the survey was conducted. Also, written informed consent was obtained from mothers before they or their children were allowed to participate. The authors of this paper sought and obtained permission from The DHS program for the use of the data. The data were completely anonymous and therefore the authors did not seek further ethical clearance before their use. The data were collected by the Ghana Statistical Service and the Ghana Health Service, with technical support from ICF Macro through the MEASURE DHS programme [31].

The Ghana 2008 DHS was designed to be representative at the national, regional and rural-urban levels. The survey employed a two-stage sampling design. The first stage selected clusters from a master sampling frame constructed from the 2000 national population and housing census. The second stage selected households from these clusters. All women aged 15–49 and all men aged 15–59 in the selected households were eligible to participate in the surveys. Three questionnaires were used for the data collection: the Household Questionnaire, the Women’s Questionnaire and the Men’s Questionnaire. At the household level, information was collected on characteristics such as source of drinking water, type of toilet facility, type of cooking fuel, and household assets. At the individual level, questionnaires were administered to one eligible woman aged 15–49 per household and one eligible man aged 15–59 per household (both randomly selected in case of multiple eligible household members), to gather information on individual characteristics and health behaviour, and information on their children. We used data on children 6–36 months old and their mothers. The total sample available in the 2008 Ghana DHS dataset was 2992. Using stratification, a total of 1411 mothers and their index child aged 6–36 months were extracted. Data on food group intake was available for 1187 dyads of mothers and their index child, which was used in the subsequent analysis. The mothers reported their children’s food consumption as well as their own food consumption.

### Ethics statement

The DHS project obtained ethical clearance from the Ghana Health Service ethical review committee before the survey was conducted. Also, written informed consent was obtained from mothers before they or their children were allowed to participate. The authors of this paper sought and obtained permission from The DHS program for the use of the data. The data were completely anonymous and therefore the authors did not have to seek further ethical clearance before their use.

### Measures

#### Child dietary diversity (DD)

The child DD score, which was the outcome variable in this analysis, was created based on the mother’s recall of the child’s consumption of food groups, 24 hours preceding the DHS interview of the mother. The mother reported whether or not the child consumed the following food groups/types: 1) Tinned, powdered or fresh milk; 2) Bread, rice, noodles, other made from grains; 3) Potatoes, cassava, or other tubers; 4) Eggs; 5) Meat (beef, pork, lamb, goat, chicken, etc.); 6) Pumpkin, carrots, squash (yellow or orange inside); 7) Any dark green leafy vegetables; 8) Vitamin A fruits; 9) other fruits containing vitamin A; 10) Liver, kidney, heart, other organs; 11) Fish or shellfish (fresh or dried); 12) Food made from beans, peas, lentils, nuts; 13) Cheese, yogurt, other milk products; 14) Oil, fats, butter, products made of them; 15) Chocolates, sweets, candies, pastries etc. The response options were ‘yes, consumed’ (score 1) and ‘no, not consumed’ (score 0). These were summed up to create the child DD score, which ranged from 0–15, and which was treated in the analysis as a continuous variable.

#### Maternal dietary diversity (DD)

The maternal DD score was also constructed based on 24-hour recall, using the same food groups/types. The maternal DD was also used in the analysis as a continuous variable.

#### Maternal and child demographic factors

Three maternal and child level demographic factors were used in the analysis: maternal age, child age and child sex. Maternal and child age were treated in the analysis as continuous variables.

#### Socio-demographic factors

Socio-demographic factors were measured at both the maternal and the household levels. The maternal level factors included education, literacy level, antenatal attendance (ANC), occupation, and empowerment. Three indices of women’s empowerment developed based on DHS recommended procedure [32] were used: (1) the number of household decisions in which the woman participates; (2) the number of reasons that justify wife beating in the respondent’s opinion; and (3) the respondent’s opinion on the number of circumstances under which a wife is justified in refusing to have sexual intercourse with her husband.

The household factors were Wealth Index, number of children under five in the household and place of residence. Some of these factors were recoded to preserve sample size and to make the findings more interpretable. Maternal education was recoded into ‘no education, primary and secondary+’ while maternal occupation was recoded into ‘white collar and agriculture/labour’. Antennal attendance was recoded into ‘none, 1–3 visits, and 4 or more visits’. The three empowerment indicators were coded ‘more empowered’ or ‘less empowered’.

### Data analysis

The analysis was performed using IBM SPSS version 21. Descriptive analysis was performed to examine the characteristics of the sample. The SPSS descriptives command was used to estimate the arithmetic means and standard deviations of continuous variables, while proportions were estimated using frequencies. Bivariate analyses were conducted to examine the associations between child DD and the various factors at maternal, child and household levels. Only factors that were significantly associated with child DD in the bivariate analyses were used in the multiple regression analysis (plus child sex). The multiple linear regression method was used to conduct the analysis, since the multiple regression method is appropriate when outcomes are continuous. The regression analysis involved the construction of three models. The first model examined the relationship between child DD and maternal and child demographic factors (child age, sex and maternal age). The second model included these socio-demographic factors: maternal education, occupation, literacy, empowerment, antenatal attendance, household wealth index, number of children under five years and place of residence, adjusting for the demographic factors. In final model, maternal DD was included, adjusting for the demographic and the socio-demographic factors. An association was considered statistically significant when p < .05. To adjust for the design effect parameters, (sample weight, strata and cluster) the General Linear Model in the SPSS Complex Samples command was used to perform the analysis. Multicollinearity was investigated and not observed [32].

## Results

### Characteristics of the sample

Tables 1 and 2 present the descriptive statistics of the sample. The mean age of children in the sample was approximately 20 months, while that of the mothers was 29 years (Table 1). About 51% children in the sample were boys. Thirty-seven percent of the mothers had no education, 24% had primary education and 39% had more than primary education (Table 2).

**Table 1:** Selected characteristics of the study sample (n = 1187), continuous variables, means and standard deviations (SDs).

Variables	Means ± SD
Child age (months)	19.80±8.55
Maternal age (years)	29.18±6.77
Child dietary diversity score	4.67±2.98
Maternal dietary diversity score	5.45±2.28
No. of children < 5 years	1.88±1.00
Participation in decision making score	2.47±1.52
Justified for refusing sex score	2.43±0.84
Wife beating not justified score	3.85±1.56

Means ± SD = values are means and standard deviations.

**Table 2:** Selected characteristics of the study sample (n = 1187), categorical variables, percentages (%).

*Variable*	*Percentage (%)*
Child sex	50.5
Male	
Female	49.5
Maternal education	
No education	36.8
Primary	24.1
Secondary+	39.0
*Antenatal attendance*	
None	4.3
1–3 visits	17.7
More than 4 visits	78.0

### Maternal and child dietary patterns

Table 3 presents the descriptive analysis of child and maternal DD. Grains were the most commonly food group consumed by mothers (86%) and children (75%). More than half of both mothers and children also consumed other vitamin A fruits and fish. Vitamin A fruits, organ meat and dairy products were the least consumed food groups by both mothers and children (Table 3). Mothers consumed more food groups (mean DD = 5.45+2.28) than did children (mean DD = 4.67±2.98) (Table 1).

**Table 3:** Food groups/types used in creating the maternal and child dietary diversity (DD).

Food type/group	Maternal DD	Child DD
	(%)	(%)
Tinned, powdered or fresh milk	15.8	19.2
Bread, rice, noodles, other made from grains	86.2	75.0
Potatoes, cassava, or other tubers	63.8	44.7
Eggs	18.7	22.2
Meat (beef, pork, lamb, goat, chicken, etc)	28.4	19.1
Pumpkin, carrots, squash (yellow or orange inside	16.3	11.6
Any dark green leafy vegetables	54.7	42.3
Vitamin A fruits	9.6	8.7
Other vitamin A fruits	63.9	53.3
Liver, kidney, heart, other organs	9.3	7.4
Fish or shellfish (fresh or dried)	72.1	56.5
Food made from beans, peas, lentils, nuts	27.8	22.7
Cheese, yogurt, other milk products	7.1	7.6
Oil, fats, butter, products made of them	53.2	44.0
Chocolates, sweets, candies, pastries, etc	16.1	32.4

% = Percentages.

### Bivariate analysis

Maternal DD was significantly associated with child DD (t = 25.26, P < .001) (not shown in Tables). Other variables that were positively associated with child DD included child age, maternal age, maternal education (secondary+), occupation, literacy, the three empowerment indicators, antenatal attendance, Wealth Index and place of residence. The number of children under five years in the household was negatively associated with child DD (t = -5.55, p < .001).

### Multiple linear regression analysis

The results of the multiple linear regression analysis are presented in Table 4. The results are presented in three models: A, B and C. In model A, only child age was significantly associated with child DD. For each month increase in age, child DD increased by 0.05 (95% CI: 0.03, 0.07). In model B, a woman’s opinion about the legitimacy of wife beating was significantly associated with child DD. A unit increase in this empowerment indicator (in the direction of disapproval) was associated with 0.14 units increase in child DD (95% CI: 0.02, 0.26). Maternal antenatal attendance was negatively associated with child DD in this analysis. In the final model (model C), wherein maternal DD was included, a statistically significant positive association between child and maternal DD was observed. A difference of one food group in mother’s consumption was associated with a difference of 0.72 food groups in the child’s food consumption (95% CI: 0.63, 0.82). Child age, maternal opinion on wife beating and antenatal attendance remained statistically significant predictors of child DD in model C.

**Table 4:** Multiple linear regression analysis of the association between maternal and child DD.

	Model (A)			Model (B)			Model (C)		
Variables	Coefficients	95% C.I	*P*	Coefficients	95% C.I	*P*	Coefficients	95% C.I	*P*
**Maternal and child basic factors**									
Child age (months)	.05	0.03, 0.07	.001	.08	0.06, 0.11	.001	.08	0.06, 0.10	.001
Child sex (female)	.34	-0.03, 0.71	.070	.30	-0.05, 0.64	.090	.23	-0.04, 0.50	.096
Maternal age (years)	.02	-0.01, 0.05	.190	-.01	-0.02, 0.031	.770	.01	-0.01, 0.03	.170
**Socio-demographic factors**									
*Maternal education*									
No education (reference)									
Primary				-.04	-0.52, 0.44	.870	-.23	-0.65, 0.18	.270
Secondary+				-.04	-0.62, 0.54	.890	.03	-0.46, 0.51	.910
*Maternal occupation*									
Agriculture/labour (reference)									
White collar				-.29	-0.71, 0.13	.180	-.11	-0.44, 0.22	.500
*Maternal literacy*									
Cannot read a word (reference)									
Can read part or whole sentence				-.29	-0.81, 0.22	.260	.10	-0.33, 0.53	.650
*Maternal empowerment*									
Participation in decision making				.03	-0.01, 0.16	.660	-.03	-0.13, 0.07	.550
Justified for refusing husband sex				.01	-0.11, 0.31	.350	.01	-0.16, 0.17	.930
Wife beating not justified				.14	0.02, 0.26	.023	.22	0.11, 0.32	.001
*Antenatal attendance*									
None (reference)									
1–3 visits				-2.16	-2.94, -1.37	.001	-2.28	-2.91, -1.64	.001
More than 4 visits				-2.94	-3.64, -2.23	.001	-2.75	-3.38, -2.11	.001
*Household wealth index*									
Poorest (reference)									
Poor				.03	-0.46, 0.52	.890	-.07	-0.45, 0.31	.720
Middle				.02	-0.62, 0.67	.950	-.15	-0.71, 0.40	.590
Rich				-.08	-0.76, 0.61	.830	.10	-0.50, 0.70	.740
Richest				-.86	-1.71, -0.01	.049	-.21	-0.89, 0.46	.540
*Place of residence*									
Rural (reference)									
Urban				-.25	-0.74,0.24	.320	-.18	-0.59, 0.23	.390
No. of children < 5 years				.01	-0.29, 0.30	.970	-.08	-0.25, 0.10	.380
**Maternal dietary diversity**									
Maternal dietary diversity score							.72	0.63, 0.82	.001
**Adjusted R squared**	**.03**			**.21**			**.48**		
**Adjusted F**	**8.80**			**9.93**			**42.78**		

DD = Dietary diversity, CI = confidence intervals.

## Discussion

This study was set out to investigate the relationship between maternal and child DD in Ghana, and the results suggest a strong positive association. An increase in maternal DD was associated with a significant increase in child DD, after accounting for the influence of child, maternal and household level factors. This implies that promoting maternal consumption of a variety of foods could improve the DD of their children (among other family members). These findings are consistent with the few previous studies that observed significant associations between maternal and child DD in both developing [27, 28] and developed countries [24, 25, 33–35]. Using data from three developing countries (Ghana, Haiti and Cambodia), researchers observed a significant association between maternal DD and minimum child DD (consumption of at least four food groups) [28]. Comparable findings were obtained in Ethiopia, Vietnam and Bangladesh, where increments in maternal DD were associated with large increases in percentage of children meeting their minimum DD requirement [27]. A related study in Southampton observed that the key influence on the quality of the children’s diets was the quality of their mother’s diets [33]. Mothers in this study who had better quality diets that complied with dietary recommendations were more likely to have children with comparable diets [33]. A systematic review concluded that parental modelling and intake are consistently and positively associated with children’s fruit, juice and vegetable intake [36]. These studies, in concert with the present research, suggest that mothers/parents play a significant role in shaping their children’s eating habits, by determining the variety and quantity of food fed to their children [37] or through parents’ own food-related behaviours and parental eating patterns [26].

Our analysis of the maternal and child dietary patterns shows that not all the food groups consumed by mothers are given to children. Even though, grains, fish and other fruits containing vitamin A, are commonly eaten by both mothers and children in this sample, mothers eat from more foods groups than do their children. This is reflected in the mean DD, where mothers have a higher mean DD than their children. This suggests that mothers select a subset of foods in the family pot to feed their children. This could be due to beliefs that certain foods are not appropriate for children, or that certain diets promote their children’s health. For example, some mothers believe that vegetables are difficult to digest and therefore can cause stomach upset in children [38, 39]. Another study revealed how some mothers used monitoring and restriction to control their children dietary intake, with the aim of ensuring the general health of the children [40].

Another plausible explanation for the differences in maternal and child dietary patterns could be cultural believes and practices outside the arena of nutritious eating per se [18]. In a study in Nigeria, parents were observed to use food restrictions to mould their children’s moral character [20]. Oniang’o and Komokoti [21] describes how food taboos negatively affect children’s dietary intake in Eastern Kenya. The above discussion suggests that interventions to improve child dietary intake should take into consideration the cultural beliefs and practices of the population. As DD is as important to the healthy development of children as it is to adults, public health and health education efforts might be improved via focused behaviour change communication having this straight-to-the-point message: young children should share the entire family pot—it is the easy and the right thing to do.

This study has both strengths and weaknesses. An important strength is the use of a nationally representative sample to investigate the relationship between child and maternal DD, permitting our findings to be generalized to mothers and children in the whole of Ghana. Additionally, the use of the same food groups for both mothers and their children makes it possible for a direct comparison between maternal DD and child DD. However, some of the other research we cite used different DD measurement methods that were specific to mothers and to children, thereby limiting direct comparison. An important limitation to this analysis has to do with the fact the data used for this paper are from a cross sectional survey. The analyses have not been able to disentangle potential reciprocal and otherwise complex causal relationships between child and maternal DD (a very simple example of reverse causation is the mother who eats a food intended for children, only to encourage a reluctant eater). The conclusions in this paper are therefore restricted to statements about the associations between the explanatory variables and the outcome variable. Another limitation worth mentioning is the 24-hour recall used in collecting the dietary data. The main weakness of this method is that the individual may not be able to report food consumption accurately due to cognitive challenges such as lack of knowledge, forgetfulness and interview situations [41]. There is evidence that the 24-hour recall tends to underestimate food intake by about 10% relative to observed intake [42]. However, cognitive challenges can be overcome by the use of probes by the interviewer, which has been well established as an effective means to identify foods that the respondent has not initially reported [41]. The DHS has made interviewer probing a key part of their interviewing protocols.

This study did not consider the effect of breastfeeding on Child DD. However, the literature suggests that breastfeeding has an influence on a child’s diet. For example, a study that investigated the effect of breastfeeding on child DD, observed that children who were heavily breastfed were more likely to have lower DD than those who were not heavily breastfed [43]. In a related study, children who were completely weaned were found to have higher DD than those who were still breastfeeding [44]. These studies highlight important differences in DD between breastfed and non-breastfed children. This is an important issue that should have been considered in the analysis of the present study.

The differences noted in the preceding paragraph notwithstanding, there are convincing evidence that breastfed children consume the same food groups as their mothers. A study using data from Ghana, Haiti and Cambodia, observed that when mothers ate a food group, breastfed children in Ghana are 7 and 19 times more likely to eat that food group [28]. Similar trends were also found in Cambodia [28]. The study further revealed that breastfeeding mothers with higher DD have children with higher DD. This suggests that irrespective of children’s breastfeeding status, they consume the same food groups as their mothers.

## Conclusions

The objective of this study was to investigate the relationship between child and maternal DD, and the results show a strong association after accounting for covariates. However, longitudinal research is needed, to trace causal relationships between the two. The results also show that in Ghana, not all the foods that are consumed by mothers are given to children. Behaviour change interventions should emphasize the importance of feeding the child all family foods, as early as possible in the first 1000 days of the child’s life.
